# Population genomics of *Staphylococcus pseudintermedius* in companion animals in the United States

**DOI:** 10.1038/s42003-020-1009-y

**Published:** 2020-06-05

**Authors:** Joshua T. Smith, Sharlene Amador, Colin J. McGonagle, David Needle, Robert Gibson, Cheryl P. Andam

**Affiliations:** 1 University of New Hampshire, Department of Molecular, Cellular and Biomedical Sciences, Durham, NH 03824 USA; 2 New Hampshire Veterinary Diagnostic Laboratory, Durham, NH 03824 USA

**Keywords:** Pathogens, Genome evolution

## Abstract

*Staphylococcus pseudintermedius* is a commensal bacterium and a major opportunistic pathogen of dogs. The emergence of methicillin-resistant *S. pseudintermedius* (MRSP) is also becoming a serious concern. We carried out a population genomics study of 130 clinical *S. pseudintermedius* isolates from dogs and cats in the New England region of the United States. Results revealed the co-circulation of phylogenetically diverse lineages that have access to a large pool of accessory genes. Many MRSP and multidrug-resistant clones have emerged through multiple independent, horizontal acquisition of resistance determinants and frequent genetic exchange that disseminate DNA to the broader population. When compared to a Texas population, we found evidence of clonal expansion of MRSP lineages that have disseminated over large distances. These findings provide unprecedented insight into the diversification of a common cutaneous colonizer of man’s oldest companion animal and the widespread circulation of multiple high-risk resistant clones.

## Abstract (web summary)

Smith et al. compare the genomes of 130 clinical *Staphylococcus pseudintermedius* isolates from dogs and cats in the New England region of the United States with those from a Texas population. They find clonal expansion of methicillin-resistant *S. pseudintermedius* lineages over large distances, which suggests the diversification of a common cutaneous colonizer of man’s companion animals.

## Introduction

*Staphylococcus pseudintermedius* is a commensal bacterium of the skin and mucous membrane and is frequently found in companion animals. Carriage of *S. pseudintermedius* often reaches >80% in some populations of healthy dogs^1^. It is also an opportunistic pathogen responsible for severe and necrotizing infections, and is frequently isolated in the skin, ears, bones, and post-surgical abscesses^1^. *S. pseudintermedius* has been found in other animals such as cats and horses, although they are not considered its natural hosts^2^. The bacterium shares several features with *Staphylococcus aureus*, the most important staphylococcal species in humans, including the capacity to express a range of virulence factors such as coagulase and other proteolytic enzymes, as well as a variety of toxins such as haemolysins, exfoliative toxins, enterotoxins, and leucotoxins^1,3^. The emergence of methicillin-resistant *S. pseudintermedius* (MRSP) is becoming a serious concern in veterinary medicine^4–6^ and highlights the need for accurate long-term surveillance. Furthermore, there have been recent reports of *S. pseudintermedius* being isolated in human carriage and infections, mainly associated with contacts with dogs (e.g., pet owners, veterinary staff)^7,8^. Although traditionally *S. pseudintermedius* has not been considered a risk for humans, recent reports have suggested that it is emerging as a zoonotic diagnosis and it may have been previously misidentified as *S. aureus* in human infections^9,10^.

Microbial population genomics involves sequencing the genomes of hundreds or even thousands of closely related strains within and between environments^11^. It has been instrumental in revolutionizing the epidemiology, surveillance and control strategies of infectious diseases that threaten human health, and has been widely used to investigate common bacterial species such as *S. aureus*, *Klebsiella pneumoniae* and *Streptococcus pneumoniae*^12^. Remarkable levels of genomic variation have been widely reported in bacterial pathogen populations, which have critical implications in understanding the origins of highly virulent and resistant lineages, successful colonization, transmission, and instances of host switching^13–15^. However, the application of large-scale whole-genome sequencing of bacterial species that naturally inhabit animal hosts remains limited. Even more problematic is when strains with multiple resistant phenotypes become prevalent in the population, but the lack of precise diagnostic and surveillance techniques makes them invisible to clinical and epidemiological studies. This gap in our knowledge ultimately limits our understanding of the underlying genetic characteristics of bacterial lineages that cause infections in animals and impacts the likelihood of precisely identifying new and emerging clones, including potential zoonoses.

The few genomic studies of *S. pseudintermedius* have offered a glimpse of its diversity, epidemiological characteristics and antimicrobial resistance (AMR) profile. A study of 12 genomes revealed that the multidrug-resistant and MRSP phenotypes in sequence types (ST) 71 and 68 in Europe evolved through a stepwise accumulation of *mec*-carrying chromosomal cassette (SCC*mec*), transposon Tn*5405* and single-nucleotide polymorphisms (SNPs) conferring fluoroquinolone resistance^4^. In the Netherlands, *S. pseudintermedius* is dominated by STs 45, 71, and 258, and genome sequencing of 50 isolates showed different resistance profiles among members of each clone^5^. These results suggest that the AMR phenotype is acquired and maintained through both vertical inheritance and horizontal gene transfer (HGT)^5^. In New Zealand, ST 71-dominated MRSP population has undergone infrequent ancestral acquisition events of the mobile chromosomal cassette SCC*mec*, which was followed by its subsequent widespread dispersal^6^. However, genomic studies aimed at elucidating the structure and dynamics of *S. pseudintermedius* populations remain scarce. Such information will be critical to addressing the question of whether the multidrug-resistant and MRSP phenotypes of *S. pseudintermedius* are restricted to one or few clones, the factors that drive their evolution, and how the species will respond to different selective pressures. It will also help us clarify the breadth of potentially useful adaptive variants that the species possesses and the underlying mechanisms that allow it to successfully switch between hosts. In this study, we carried out a population genomics analysis of 130 clinical *S. pseudintermedius* isolates from dogs and cats across five states in the New England region of the United States sampled from 2017 to 2018. Results reveal the diversification and widespread circulation of multiple high-risk resistant clones.

## Results

### Diverse lineages are co-circulating in New England

**Fig. 1:**
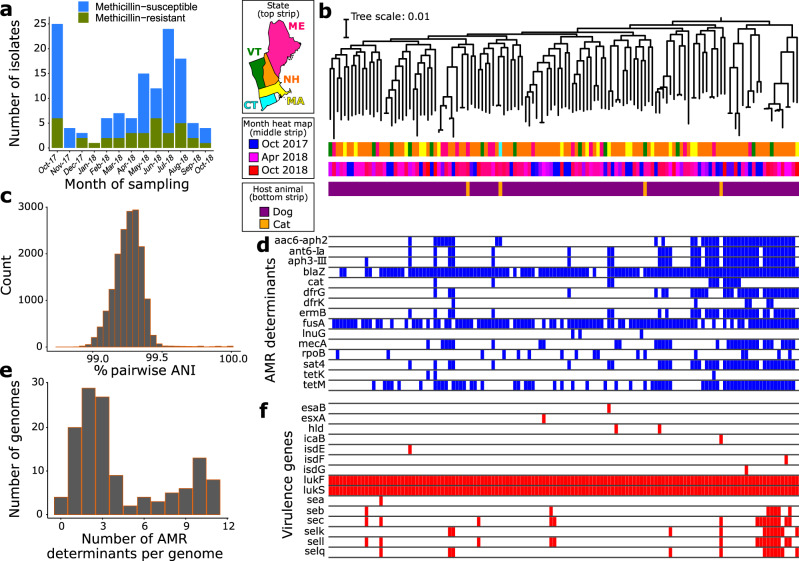
Characteristics of the New England *S. pseudintermedius* population (*n* = 130 genomes). **a** Monthly sampling of MRSP and MSSP isolates based on results of disk diffusion test. **b** The midpoint-rooted maximum likelihood phylogenetic tree was calculated using sequence variation in 1906 core genes. Scale bar represents nucleotide substitutions per site. For visual clarity, not all months of sampling are shown on the color legend of month heat map; however, all months are shown on the middle strip. **c** Frequency distribution of pairwise genome-wide ANI values. **d** Gene presence–absence matrix showing the distribution of AMR determinants present in each genome (blue blocks–present; white–absent). AMR determinants refer to both horizontally acquired genes and allelic variants of a gene. **e** Distribution of the number of AMR determinants per genome. **f** Gene presence–absence matrix showing the distribution of virulence-associated genes present in each genome (red blocks–present; white–absent). Each column in the two matrices correspond to a genome on the phylogeny. N*H* New Hampshire, *MA* Massachusetts, *CT* Connecticut, *ME* Maine, *VT* Vermont.

We obtained a total of 162 *S. pseudintermedius* isolates from routine diagnostic tests of clinical specimens submitted to the New Hampshire Veterinary Diagnostic Laboratory (NHVDL) from October 2017 to October 2018. Of the 162 *S. pseudintermedius* isolates, we retrieved high-quality draft genome sequences for 130 isolates, composed of 126 genomes from dogs and four from cats (Supplementary Fig. 1 and Supplementary Data 1). More than half of the isolates came from New Hampshire (*n* = 84), whereas the remainder were from neighboring states in New England (Massachusetts, Connecticut, Maine, and Vermont). Initial in vitro screening of methicillin and other beta-lactam antibiotic resistance was performed using disc diffusion testing. Results revealed the presence of 36 MRSP isolates and 94 methicillin-susceptible *S. pseudintermedius* (MSSP) isolates throughout the entire study period. We found MRSP isolates in almost every month of sampling, with as many as six out of 12 isolates being MRSP in a single month (Fig. 1a).

De novo genome assembly generated sequences of sizes ranging from 2.44 to 2.93 Mb (mean = 2.61 Mb) and the number of predicted genes ranged from 2260 to 2850 (mean = 2449) (Supplementary Data 1). We used Roary^16^ to estimate the pan-genome of the entire New England *S. pseudintermedius* population. Roary identified a total of 8207 orthologous gene families in the pan-genome, which can be classified into core genes (*n* = 1906 genes), soft core genes (*n* = 108 genes), shell genes (*n* = 741 genes), and cloud genes (*n* = 5452) (Supplementary Fig. 2 and Supplementary Data 2). The combined core and soft core genes comprised 24.5% of the pan-genome, whereas the combined shell and cloud genes (which together make up the accessory genome) comprised 75.5% of the pan-genome. It is notable that many accessory genes were unique to a single strain (2797 genes, representing 34.1% of the pan-genome). The large accessory genome in our results is not uncommon and has been reported in different bacterial pathogens, including *S. aureus*^17,18^ and *Staphylococcus haemolyticus*^19^. However, we recognize that pan-genome sizes and precise characterization of core and accessory genes are greatly influenced by the phylogenetic distance, sequence quality of genomes, the number of genomes being compared, assembly and annotation methods, and choice of threshold values to define orthologous genes. The maximum likelihood phylogenetic tree based on the sequence alignment of the 1906 core genes reveal many deep branching lineages that have relatively little structure relative to sampling location (state) or month of sampling (Fig. 1b). The four isolates from cats are intermingled among the dog isolates throughout the tree. To further ensure that all isolates are from the same species and determine the degree of overall genomic relatedness among the *S. pseudintermedius* isolates, we calculated the pairwise average nucleotide identity (ANI) of all orthologous genes shared between any two genomes^20^. Genome-wide ANI values for every possible pair of *S. pseudintermedius* genomes range from 98.8–100% (mean = 99.3%) (Fig. 1c, Supplementary Fig. 3 and Supplementary Data 3).

We considered bioinformatic evidence for the presence of different AMR determinants in our samples. We used ARIBA to detect the presence of horizontally acquired AMR genes and resistance alleles owing to chromosomal mutations^21^. The distribution of AMR determinants varied substantially among the genomes and we did not find evidence for phylogenetic or geographical clustering of any one of the AMR genes (Fig. 1d and Supplementary Data 4). Two mechanisms confer penicillin resistance in *Staphylococcus*: the production of beta-lactamase encoded by *blaZ*, which inactivates penicillin by hydrolysis of its beta-lactam ring, and the penicillin-binding protein PBP2a encoded by *mecA*^22^. We detected *blaZ* in 111 isolates representing 85.4% of the population, and the *mecA* gene in 32 genomes representing 24.6% of the population. The genes *tetK* and *tetM*, which confer resistance to broad-spectrum tetracyclines, were detected in three and 59 genomes, representing 2.3% and 45.4% of the population, respectively. We also detected resistance determinants for other antimicrobial classes. Many genomes carry genes that encode resistance against aminoglycosides (aac(6)-aph(2), aph(3')-III, ant(6')-Ia, sat4a; *n* = 38, 38, and 39 genomes, respectively), chloramphenicols (*cat*; *n* = 12 genomes), daptomycin (*rpoB* allelelic variant; 17 genomes), diaminopyrimidines (*dfrG*, *dfrK*; *n* =31 and 6 genomes, respectively), fusidic acid (*fusA* allelelic variant; 99 genomes), lincosamides/macrolides/streptogramin B (*lnuG, ermB*; *n* = 3 and 34 genomes, respectively), and streptothricin (sat4; *n* = 38 genomes). Only four isolates out of the 130 genomes do not contain any known AMR determinant, whether it is a resistance allele or a horizontally acquired gene. Although none of the isolates from the four cats are MRSP, all four genomes harbor DNA that encodes resistance against other antibiotic classes. Overall, we found that a total of 126 *S. pseudintermedius* genomes carry at least one AMR determinant, and a remarkable 41 genomes contain ≥5 AMR determinants per genome (Fig. 1e).

Mobile genetic elements (MGEs) play an important role in the evolution and diversity of *Staphylococcus* and facilitate the dissemination of AMR genes within and between species^23^. Here, we explored the presence and types of the chromosomal cassette SCC*mec*, which can facilitate the mobilization and distribution of *mecA* and other AMR genes in *Staphylococcus*^24,25^. SCCmec elements are highly variable in terms of their structural organization and gene content, but are classified mainly based on the *ccr* and *mec* gene complexes, the key elements of the cassette responsible for integration and excision of SCC*mec* and the beta-lactam resistance phenotype, respectively^26^. A total of 13 SCC*mec* types and numerous subtypes have been identified in *S. aureus* to date^26^. Of the 32 *S. pseudintermedius* genomes that carry the SCC*mec*, we identified three known types (Type III (also known as Type II–III in MRSP^27^), *n* = 2 genomes; Type IV, *n* = 10 genomes; Type V, *n* = 15 genomes) and five genomes with unknown type (Supplementary Fig. 4 and Supplementary Data 1). These results were similar to those identified in veterinary MRSP in Australia^27^. Their locations in the phylogenetic tree show that the MRSP phenotype has been derived from at least 13 independent acquisitions of different SCC*mec* elements. In *S. aureus*, Type III is found predominantly in hospital-associated methicillin-resistant *S. aureus* (MRSA) and carry resistance genes for cadmium, tetracycline, mercury, erythromycin, and spectinomycin, in addition to *mecA*^28^. Hence, a single transfer event of Type III can turn a susceptible strain into a multidrug-resistant strain. Types IV and V are often found in community-associated MRSA and carry only the *mecA* gene^28^. In *S. pseudintermedius*, SCC*mec* III tended to be healthcare-associated MRSP lineages, whereas isolates with SCC*mec* V tended to be community-associated MRSP lineages^29^. The five untypeable SCC*mec* highlight the need to continue efforts to discover and characterize undiscovered variants of SCC*mec* elements, especially in non-*aureus* species.

We identified several virulence genes commonly found in *S. aureus* in the New England *S. pseudintermedius* population (Fig. 1f and Supplementary Data 5). The two-component pore-forming leukocidin genes *lukF* and *lukS*^30^ were present in all genomes. The synergistic action of these genes produces a toxin that damages membranes of host defense cells and erythrocytes^3,31^. Genes associated with other virulence factors were also detected from the genomic sequences, although present in lower frequencies. These include enterotoxins encoded by *sec* (*n* = 15 genomes), *selK* (*n* = 11 genomes), *selI* (*n* = 15 genomes), and *selq* (*n* = 12 genomes). Staphylococcal enterotoxins encoded by *sec* and *sel* cause superantigenic and emetic activities in the bacterium^31^.

Overall, we found high levels of phylogenetic and genomic diversity in *S. pseudintermedius* isolates co-circulating in New England only within a single year of sampling. The population consists of several methicillin-resistant, multidrug-resistant, and virulent clones present in dogs and cats.

### Comparison of two *S. pseudintermedius* populations

**Fig. 2:**
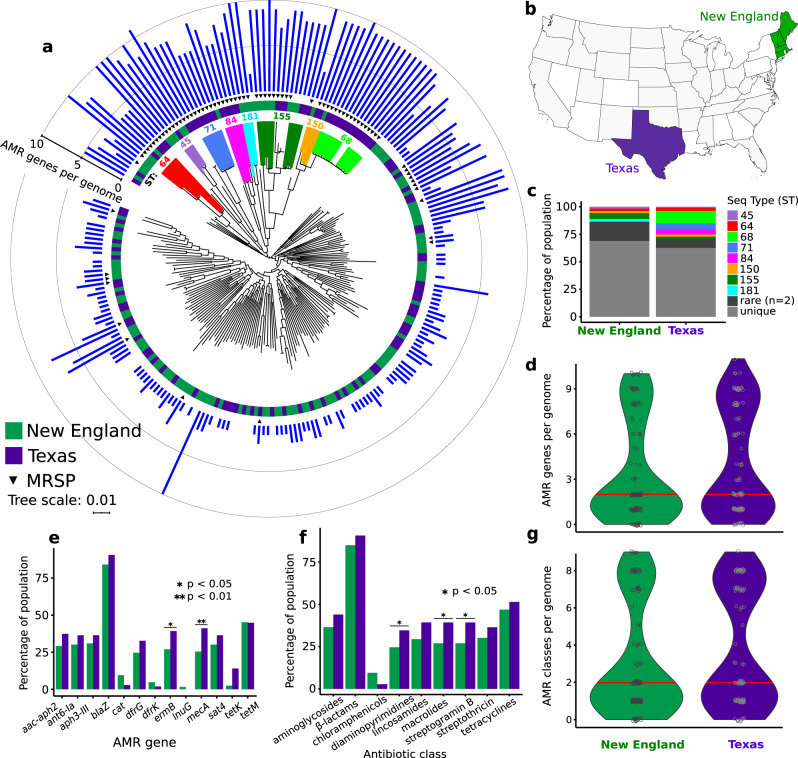
Phylogenetic relationship and AMR profiles of 126 New England genomes and 107 Texas genomes of *S. pseudintermedius*. All isolates in this tree were sampled from clinical specimens from dogs submitted to the veterinary laboratory in each region. **a** Midpoint-rooted maximum likelihood tree built using concatenated sequence alignments of 1776 core genes present in both New England and Texas populations. Scale bar represents nucleotide substitutions per site. Upside down triangles indicate the presence of *mecA* in genome sequences. Blue bar plots represent number of horizontally acquired AMR genes per genome. **b** Map of the United States showing the New England and Texas regions. **c** Distribution of STs per region. Comparison between the New England and Texas populations in terms of the total number of AMR genes per genome **d**, percentage of each population that carry specific AMR genes **e**, percentage of the population that carry at least one AMR gene for each major class of antibiotic **f**, and the total number of genomes that are resistant to zero, one or multiple classes of antibiotics **g**. Red horizontal lines in **d** and **g** represent median values. **e**, **f** Only statistically significant differences are shown for visual clarity. **g** Counts of genomes are based on the presence of at least one AMR gene that represent each major class of antibiotic.

To place our data set in a country-wide context, we compared the genome sequences of New England *S. pseudintermedius* to a previously published genomic dataset from Texas^32^ (Fig. 2a, Supplementary Fig. 3 and Supplementary Data 1). New England, located in the northeast corner of the United States, is ~2770 km from Texas, which is in the southern part of the country (Fig. 2b). We chose this data set because its sampling strategy was most similar to our study, i.e., only isolates from clinical specimens from dogs collected by the Texas Veterinary Medical Teaching Hospital and a mix of MRSP and MSSP^32^. Of the 160 Texas genomes sequenced in that study, we did not include genomes of isolates from healthy dogs and those with low-quality sequences. To minimize the confounding effect of the host animal, we also excluded the four isolates from cats in the New England data set. Overall, we compared 126 New England and 107 Texas genomes, all from disease cases in dogs.

We next re-ran Roary on each data set. The composition of the pan-genome varied between the two populations. The New England population has a total of 8122 genes (1910 core, 107 soft core, 765 shell, 5340 cloud genes), whereas the Texas population has a total of 6845 genes (1836 core, 133 soft core, 1020 shell, 3856 cloud genes) (Supplementary Data 2). The combined New England and Texas data set has a total of 9817 genes (1776 core, 166 soft core, 905 shell, 6970 cloud genes) (Supplementary Fig. 5 and Supplementary Data 2). We used the concatenated sequences of 1776 core genes that are common to both New England and Texas populations, generating a 1.60 Mb sequence alignment, to build a maximum likelihood phylogeny (Fig. 2a). We observed frequent intermingling of isolates from each population across the tree, suggesting widespread dissemination across a large geographical distance.

We carried out a second AMR analysis to compare horizontally acquired AMR genes in the two populations using ABRicate (Supplementary Data 6). In this analysis, we did not use ARIBA because ARIBA requires the raw reads as input, which were not available for the Texas genomes. Based on in silico detection of the *mecA* gene, ABRicate identified 44 MRSP and 63 MSSP in the Texas population (Fig. 2a). In both New England and Texas populations, the MRSP lineages are mostly clonal, indicated by the extremely short branches at the tip of each clonal branch. The most common MRSP STs that had representatives from both regions were STs 45, 64, 150, and 155. There is also evidence for clonal expansion of some MRSP lineages within each region. For example, STs 68, 71, and 84 were found only in Texas, whereas ST 181 was found only in New England. Moreover, in both populations, the MSSP lineages are genetically diverse and are found in particularly long branches. There were also numerous STs that were either rare (here, defined as present in only two genomes) or unique (present only in a single genome) (Fig. 2c). STs that are intermingled with another may reflect recent divergence or recombination of the MLST genes.

Many genomes of the eight most common MRSP STs and their close relatives also carry the highest number of horizontally acquired AMR genes per genome, ranging from 2 to 11 AMR genes (median = 9 AMR genes per genome) (Supplementary Data 6). We did not find significant differences between the two populations in terms of the total number of AMR genes per genome (*p* value = 0.14, Welch's *t* test) (Fig. 2d). We further queried the genomes to compare the distribution of specific AMR genes between New England and Texas (Fig. 2e). Both had comparable proportions of genomes that carry many of these AMR genes, such as *aac-aph2*, *blaZ*, and *tetM*. However, we found significant differences between the two populations in those genomes that carry the gene *ermB* (*p* = 0.0233, *z* score test) and *mecA* (*p* = 0.0054, *z* score test). For both genes, the Texas population exhibited higher number of genomes as a proportion of their respective population size. In terms of the major classes of antibiotics, both populations have high numbers of genomes that carry genes conferring resistance against beta-lactams, but they differ in the proportions of genomes that carry resistance genes for macrolides (*p* = 0.0233, *z* score test), streptogramin B (*p* = 0.0233, *z* score test) and diaminopyrimidines (*p* = 0.0475, *z* score test) (Fig. 2f). We also found that a total of 45 genomes representing 35.7% of the New England population and 48 genomes representing 44.9% of the Texas population carry resistance genes to ≥3 antibiotic classes (Fig. 2g). We did not detect any horizontally acquired AMR genes in 15/126 (11.9%) and 10/107 (9.35%) of the New England and Texas genomes, respectively.

**Fig. 3:**
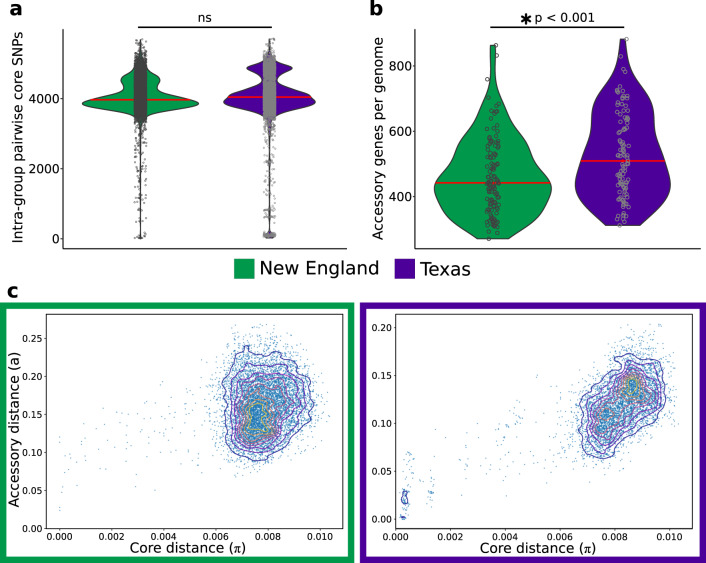
Pan-genome differences between New England (*n* = 126 genomes) and Texas (*n* = 107 genomes) populations. **a** Pairwise core SNPs. **b** Number of accessory genes per genome. Red horizontal lines represent median values. **c** Contour plots of pairwise distances between genomes in terms of their core genome divergence (measured by SNP density across the core genome) and the difference in their accessory genomes (measured by the Jaccard distance based on the variation in the gene content of their sequences).

Within-species diversity stems from allelic differences in core genes as well as gene content variation in the accessory genomes among strains^14^, which may partly stem from differential response to local selective pressures through frequent DNA gain and loss^13,14^. We therefore sought to determine to what extent the genome structures of *S. pseudintermedius* in New England and Texas have diverged. First, using the 1.60 Mb core genome alignment, we calculated the total number of SNPs between each pair of genomes from each population. We did not find significant differences in the number of SNPs in the core genome (*p* value = 0.46, Welchʼs *t* test) (Fig. 3a), but found significant differences in the number of accessory genes per genome between the two populations (*p* value < 0.00016, Welch's *t* test) (Fig. 3b). To gain insight on how the accessory genome has diverged in relation to the core genome^14^, we used PopPUNK, which employs pairwise nucleotide k-mer comparisons with distinguish shared sequence and gene content^33^. We found similar patterns in the distribution of pairwise genomic distances between New England and Texas, with larger genetic distances concentrated away from the origin (Fig. 3c). This pattern is indicative of the presence of multiple genetically distinct clusters that are diverging in both core sequences and accessory gene content. These results expand the core genome-based phylogenetic analysis described above (Fig. 2a). Overall, these data show that each of the two geographically distinct populations of *S. pseudintermedius* in the United States is composed of numerous genetically distinct lineages that can be distinguished in both their core and accessory genome divergence patterns.

### Heterogeneity in the frequency of recombination

Many bacterial species are known to receive DNA fragments from other taxa and integrate them into their genomes through recombination^34^. The process of recombination has a fundamental role in the evolution of many bacterial pathogens and has been implicated in the emergence of new phenotypic traits such as AMR, ecological adaptation, and host colonization^34^. Recombination is known to contribute to the evolution of *Staphylococcus*, as in the case of *S. aureus* and *Staphylococcus epidermidis*, which have been reported to frequently recombine within and between species^35^. Here, we sought to determine the frequency and characteristics of recombination in *S. pseudintermedius* because this process may likely contribute to its genomic variation and diversification.

**Fig. 4:**
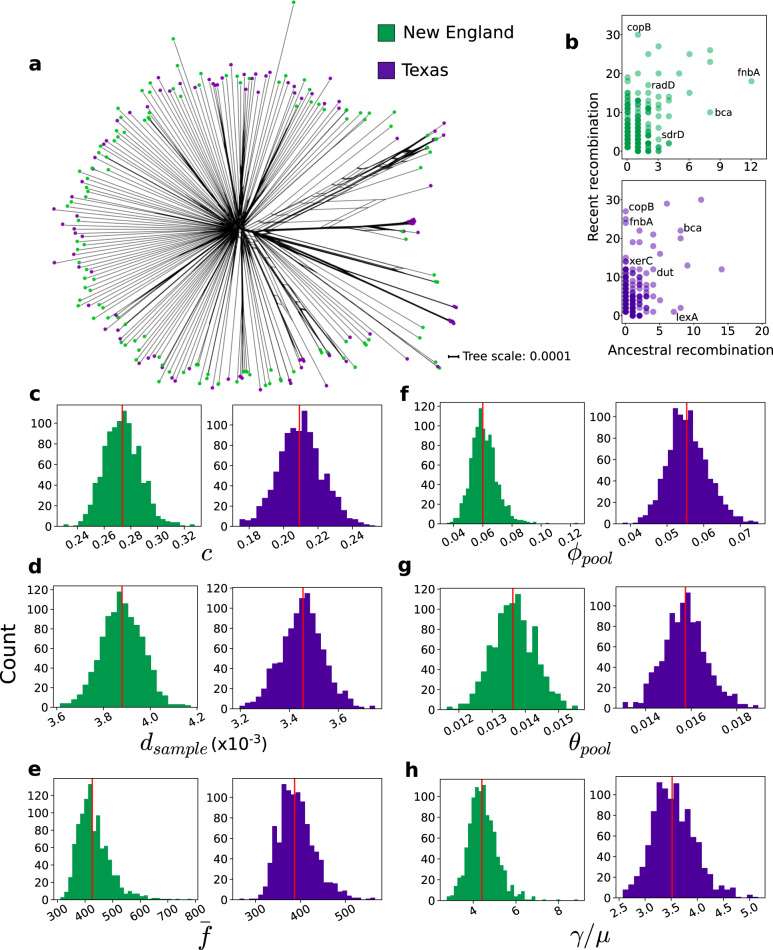
Recombination in *S. pseudintermedius* genomes from New England and Texas. **a** A phylogenetic SplitsTree network of the core genome. Scale bar represents nucleotide substitutions per site. **b** Genes that have undergone recent and ancestral recombination. Horizontal axis shows the estimated number of ancestral recombinations, and vertical axis shows the estimated number of recent recombinations. Names of some of the most frequently recombined genes with known functions are shown. List of all recombined genes are shown in Supplementary Data 7. **c**–**g** Evolutionary and recombination parameters calculated by mcorr.: **c** recombination coverage; **d** diversity; **f** mean fragment size of a recombination event; *ϕ* recombinational divergence; *θ* mutational divergence; *γ/μ* relative rate of recombination to mutation (equivalent to ratio of ϕ/θ). Red vertical line in each plot represents the inferred value of each parameter calculated with 1000 bootstrapped replicates.

We used different methods to elucidate the impact of recombination on the population structure and evolution of *S. pseudintermedius*. First, under the null hypothesis of no recombination, we calculated the pairwise homoplasy index (PHI)^36^ and detected evidence for significant recombination in the core genome of the combined New England and Texas data set (*p* = 0.0, permutation test). Using the sequence alignment of the 1776 core genes shared by the New England and Texas genomes, we used the SplitsTree network^37^ to visualize the reticulated, star-like evolutionary history of the combined *S. pseudintermedius* populations (Fig. 4a). We next sought to identify frequently recombining genes in each population by running fastGEAR^38^ on individual sequence alignments of core and shared accessory genes. The program fastGEAR identifies both recent (i.e., recombination events affecting a few strains) and ancestral (i.e., recombination events affecting entire lineages) recombinations (Fig. 4b, Supplementary Data 7). In the New England population, we found that a total of 961 genes have had a history of recombination. A total of 922 and 199 genes were involved in recent recombination and ancestral recombination, respectively. In contrast, we identified a total of 869 genes that have experienced recombination in the Texas population, with 799 and 184 genes that have experienced recent and ancestral recombination, respectively. Although many of the frequently recombined genes have hypothetical or unknown functions, we found that *copB*, which has a role as a copper-translocating P-type ATPase^39^, is frequently recombined in both populations. In *S. aureus*, this gene is part of the operon that promotes copper hypertolerance and enhanced resistance to phagocytic killing^39^. Metal resistance genes, including *copB*, are carried by some SCC*mec* variants in both human and animal MRSA and other species of *Staphylococcus*^40^. Hence, *copB* is likely co-translocated and/or co-selected with AMR genes in the SCC*mec*. Another gene that has experienced frequent recombination is *fnbA*, which encodes a fibronectin-binding protein and thus promotes colonization to different anatomical sites of the eukaryotic host^41^. The gene *bca*, which encodes the surface-associated C protein alpha antigen^42^, is also frequently recombined in both populations. In *S. aureus*, sequence variation in *fnbA* has been previously reported to contribute to differences in antigenicity between strains^41,43^.

We also wanted to compare different genome-wide evolutionary and recombination parameters between New England and Texas populations. We used the core genome alignment of each data set as an input to mcorr^44^ (Fig. 4c–g and Supplementary Data 8). The recombination coverage (c), which indicates the fraction of the genome whose diversity was derived from recombination events since the last common ancestor of the population, was estimated to be 0.274 (standard deviation [sd] = 0.014) and 0.210 (sd = 0.013) in New England and Texas, respectively. Recombination coverage ranges from 0 (clonal evolution) to 1 (complete recombination)^44^. These values indicate that 27.4% and 21.0% of sites in any one genome from New England and Texas, respectively, originated from recombination events. The diversity (*d*) is the probability that a pair of genomes will differ at any locus and is estimated from the diversity generated from both recombination and accumulation of mutations of the clonal lineage^44^. This parameter was estimated to be 3.88 × 10^−3^ (sd = 8.80 × 10^−5^) and 3.46 × 10^−3^ (sd = 8.73 × 10^−5^) in New England and Texas, respectively. The mean fragment size (*f̅*) of a recombination event was estimated to be 426.14 (sd = 60.22) bp and 387.09 (sd = 40.55) bp in New England and Texas, respectively. Recombinational divergence (*ϕ*) was estimated to be 0.060 (sd = 0.009) and 0.055 (sd = 0.006) in New England and Texas, respectively. The mutational divergence (*θ*), which refers to the mean number of mutations per locus since divergence of a pair of homologous sites in the pool, was estimated to be 0.014 (sd = 6.39 × 10^−4^) and 0.016 (sd = 9.48 × 10^−4^) in New England and Texas, respectively. Lastly, the ratio *ϕ*/*θ* (or γ/μ), which gives the relative rate of recombination to mutation, was estimated to be 4.40 (sd = 0.683) and 3.52 (sd = 0.413) in New England and Texas, respectively. For all parameters, we found significant differences between the two populations (*p* value <0.001 for all six parameters; Welch's *t* test). For comparison, *S. aureus* has lower values for recombination divergence (0.042) and *ϕ*/*θ* (1.0), and higher values for recombination coverage (0.36), diversity (0.015), mean fragment size (550 bp), and mutational divergence (0.042)^44^. Overall, we found that heterogeneity in rates and characteristics of recombination has contributed to shaping the genomic structure and diversity of the New England and Texas populations of *S. pseudintermedius*.

## Discussion

We present a comparative population genomics study of *S. pseudintermedius* in the United States. Results revealed the co-circulation of phylogenetically diverse lineages that have access to a large pool of accessory genes, including AMR genes. For *S. pseudintermedius*, the long-term clinical and public health significance of MRSP and multidrug-resistant lineages remains unclear. Suffice to say that companion animals can act as a reservoir of resistant strains that can be transmitted to humans or other animals, as has been reported for other *Staphylococcus* species that colonize multiple eukaryotic hosts^45,46^. *S. pseudintermedius* also appears to be undergoing a considerable amount of genetic exchange, which can lead to the emergence of high-risk clones and those with hitherto uncharacterized virulence capacity. The origins of recombined and horizontally acquired DNA remains unclear, but they were likely derived from other *S. pseudintermedius* strains, other *Staphylococcus* species that they co-inhabit their niche with, and even distantly related organisms. This has been demonstrated in a recent study that reported that many resistance genes have been frequently exchanged between different *Staphylococcus* species and between *Staphylococcus* and other non-*Staphylococcus* taxa^47^. The ability of *S. pseudintermedius* to take up and integrate large DNA segments highlights the pathogen's capacity for rapid evolution and adaptation to primary and secondary hosts, similar to what has been observed in *S. aureus*^35,46^. Frequent recombination may also contribute in part to the pathogenicity of *S. pseudintermedius* and its ability to colonize other host species. Recombination is not the only process that contributes to pathogen diversity. Extensive phylogenetic intermixing of strains spanning a large geographical distance suggests substantial translocation and/or exchange of strains. This may likely be a result of either widespread pet transport and distribution across the country or that there is a country-wide pool of circulating MSSP and MRSP lineages, with indication of local expansion and diversification of certain MRSP clones in each region.

A few limitations need to be acknowledged. The retrospective nature of the isolate collection we used and sampling bias owing to the inclusion of only clinical submissions mean that sampling may have missed some less-prevalent genotypes and counties in New England. We also did not have access to data regarding the antibiotic history, treatment received, or clinical outcomes of the dogs and cats from which isolates were obtained. Another limitation is our use of databases for querying AMR genes, virulence genes and SCC*mec* types. These databases were developed primarily for *S. aureus*, of which thousands of genomes have already been sequenced (for example, see ref. ^46^). Less is known of other *Staphylococcus* species, however, and this gap in current knowledge may have important consequences. For example, newly discovered genetic variants (as in the case of the five unknown SCC*mec* types) may be present in other species but remain invisible from current in silico detection methods and databases, which make them difficult to track over the long term. This also means that clinical decisions and treatment options made when treating diseases caused by non-*aureus* species are based on assumptions from studies of *S. aureus*. Estimates of evolutionary and recombination parameters show considerable differences not only between the two *S. pseudintermedius* populations but also between *S. pseudintermedius* and *S. aureus*, which can impact population dynamics and the emergence of favorable phenotypes. Hence, more extensive and large-scale genome sequencing of non-*aureus* species will greatly augment this gap in our knowledge of the diversity, pathogenicity, and resistance mechanisms of other *Staphylococcus* species that can potentially develop into formidable high-risk pathogens. Notwithstanding these limitations, we obtained sufficient representation of the diversity of *S. pseudintermedius* and the widespread distribution of MRSP and multidrug-resistant clones even within a few years of sampling, which was the main focus of this study.

The results presented here open multiple avenues for future research. First, the integration of genomic epidemiology in veterinary medicine will advance our ability to identify targets for strain differentiation, surveillance, and diagnostics. It is imperative that long-term monitoring of the eight major STs with the highest number of horizontally acquired AMR genes (STs 45, 64, 68, 71, 84, 150, 151, and 181) should be carried out, as they appear to be expanding in New England and Texas and likely in other parts of the country. Future work using functional assays is also needed to precisely characterize the virulence factors of *S. pseudintermedius*, instead of simply relying on *S. aureus* studies. Our results on the most frequently recombining genes (e.g., *copB*) provides an important starting point to understand the genetic basis of *S. pseudintermedius* pathogenicity. Second, local and global surveillance of resistant phenotypes is particularly critical to detect and limit the emergence of new resistant clones with the capacity to spread and switch between animals and humans. Future work should include determining the prevalence of MRSP and multidrug-resistant strains across a range of domestic and wildlife animals, and specific groups of people who are in constant proximity to animals (e.g., veterinary personnel, pet owners, breeders, pet shop owners, pet adoption workers, farmers). Close contact between animals and humans has facilitated multiple host-switching events in *S. aureus*^48–50^ and it is not unreasonable to assume that it can also occur in *S. pseudintermedius*. Investigation into the extent of host switching (i.e., animal-to-human and between dogs and other animal species) and the adaptive changes associated with radical changes in host ecology will reveal key host–pathogen interactions that could be targeted for novel clinical interventions and therapies. Third, the precise origins of MRSP (whether from human-associated methicillin-resistant *S. aureus*, methicillin-susceptible animal-associated *S. pseudintermedius* or other staphylococcal species) remains unclear and genomic sequencing approaches will certainly advance our understanding of how and when it emerged in companion animals. We also encourage the application of population genomics as an integral component of the One Health initiative, which emphasizes that the health of humans, animals, and the environment are inextricably linked^51^. Taken together, our findings provide a genomic framework that will provide a critical foundation and practical support for future studies, investigating the population structure and dynamics, drug resistance and diversification of *S. pseudintermedius* as well as the risks to pets, pet owners, and veterinary personnel. It will facilitate more deeply informed genomic tracking and surveillance for the emergence and convergence of virulence and AMR in certain genotypes of *S. pseudintermedius* and in different geographical regions. In summary, we underscore the value of elucidating the population genomic structure and evolution of this increasingly important pathogen to advance the health of man’s favorite companion and oldest domestic animal.

## Methods

### Bacterial sample collection

The *S. pseudintermedius* isolates were acquired as culture swabs from routine clinical specimen submissions to NHVDL, Durham, New Hampshire, USA, from October 2017 to October 2018. The clinical specimens were received from veterinary practices in the New England region that includes the states of Connecticut, New Hampshire, Maine, Massachusetts, and Vermont. No live vertebrates were used in this study; therefore, the NHVDL is exempt from the IACUC approval process. All isolates were from disease cases. Pure isolates of *S. pseudintermedius* were cultured on commercially prepared tryptic soy agar with 10% sheep red blood cells. Initial species identification was carried out using matrix-assisted laser desorption/ionization time-of-flight mass spectrometry (MALDI-TOF MS), which has been previously demonstrated to be a sensitive and specific species-level identification tool for *S. pseudintermedius*^6,32^. We used the Bruker Biotyper MALDI-TOF MS following manufacturer’s protocols. The addition of formic acid in the extended direct technique was used when necessary to ensure a reliable log(score), which refers to the level of similarity between an unknown tested specimen and a reference sample. Species assignments were made when log(score) values were ≥2.0 and identical species were included in the top two database matches when compared with the library of reference spectra available in the Bruker MBT Compass, RUO library 6903(V6) and 7311(V7) (Bruker Daltonics, Bremen, Germany). The most common sites of isolation included skin, ears, urine, and wounds. Metadata information of isolates are displayed in Supplementary Data 1. All isolates were stored in DMSO solution in −80 °C.

### Methicillin susceptibility screening

Isolates were initially screened in vitro at the NHVDL with the Kirby Bauer disc diffusion technique using both the cefoxitin and oxacillin discs. Whereas cefoxitin predicts methicillin resistance in *S. aureus*, oxacillin is used as the official predictor of methicillin resistance for S. *pseudintermedius* following breakpoint guidelines of the most current Clinical and Laboratory Standards Institute^52^. For methicillin-resistant isolates, we further tested for the presence of the penicillin-binding protein PBP2 using a commercial latex agglutination test (MASTALEX MRSA Latex Kit, MAST, UK) following manufacturer's protocols. Verification for the presence of the *mecA* gene was done using whole-genome sequencing (described below).

### DNA extraction and whole-genome sequencing

Cultures were grown in brain heart infusion broth at 37 °C for 24–48 h. DNA was extracted and purified from cultures using the Zymo Research Quick-DNA Fungal/Bacterial Miniprep Kit (Irvine, California) following manufacturer's protocols. DNA concentration was measured using a Qubit fluorometer (Invitrogen, Grand Island, NY). DNA libraries were prepared using the Nextera XT protocol (as per the manufacturer's instructions) with 1 ng of genomic DNA/isolate. Samples were sequenced as multiplexed libraries on the Illumina HiSeq platform operated per the manufacturer's instructions to produce paired end reads of 250 nucleotides in length. Sequencing was done at the UNH Hubbard Center for Genome Studies, Durham, NH, USA.

### Genome assembly, annotation, and pan-genome analysis

Reads were assembled into contigs using the de novo assembler SPAdes v.3.13.1^53^. The resulting contigs were annotated using Prokka, a stand-alone tool specifically developed for annotating bacterial genomes^54^. Genome assembly quality was assessed using Quast^55^. Sequencing failures and poor overall sequence qualities are known to occur with any sequencing platform. We used CheckM to assess the quality of our sequences and excluded genomes with <90% completeness and >5% contamination^56^. After filtering out the genomes with low coverage and of poor quality and exclusion of any assemblies with >200 contigs and an N50 <40,000 bp, we used a total of 130 genomes for downstream analysis, with the numbers of contigs ranging from 25 to 196 and N50 ranging from 50,676 to 351,605 bp (Supplementary Fig. 1 and Supplementary Data 1). We also confirmed the species identity using a BLASTN^57^ search of the NCBI non-redundant (nr) database using the annotated 16S rDNA sequences of each genome in our study. To determine the degree of genomic relatedness and hence clarify whether these genomes belong to the same species, we calculated the genome-wide ANI for all possible pairs of genomes using the program FastANI v.1.0^20^. ANI is a robust similarity metric that has been widely used to resolve strain relatedness and determine whether any two strains belong to the same or different species^20^. We used the program Roary^16^ to characterize the pan-genome of the 130 New England *S. pseudintermedius*. Roary iteratively pre-clusters protein sequences using CD-HIT^58^, all-against-all BLASTP^57^ and Markov clustering^59^. Roary then sorts all orthologous gene families identified in the pan-genome into four categories: core (genes found in ≥99% strains), soft core (found in 95% ≤strains <99%), shell (found in 15% ≤strains <95%), and cloud (found in >15% of strains)^16^. We used the default parameters for all programs used.

### Comparative population genomics of New England and Texas

We compared 126 New England genomes with 107 previously published genomes from Texas for a total of 233 genomes^32^. For both data sets, we included only those clinical isolates from dogs (disease cases). The four genomes from cats in the New England data set were excluded. A list of accession numbers and metadata for the Texas *S. pseudintermedius* genomes were included in Supplementary Data 1. To maintain consistency in gene annotations, we used Roary to characterize the pan-genome of this merged data set and re-annotated them using Prokka with default parameters^54^. The US map used in the design of Figs. 1 and 2 was obtained from https://simplemaps.com.

### Phylogeny and population structure analyses

For each of the two data sets (i.e., the 130 New England genomes and the 233 combined New England+Texas genomes), each single-copy orthologous gene family obtained from the Roary analysis was aligned using MAFFT^60^. The alignments were concatenated to give a single core alignment, and a maximum likelihood phylogeny was then generated using the program Randomized Axelerated Maximum Likelihood (RAxML) v.8.2.11^61^ with a general time-reversible nucleotide substitution model^62^ and four gamma categories for rate heterogeneity. Genetic population structure analysis was performed using R-implemented hierarchical Bayesian analysis of population structure (RhierBAPS) with default parameters using nested clustering and with the core genome alignment as input^63^. All phylogenies were visualized using the Interactive Tree of Life^64^. To further elucidate the population structure of *S. pseudintermedius* based on the divergence of both shared sequence and gene content in a population, we used PopPUNK (Population Partitioning Using Nucleotide *K*-mers) with default parameters^33^. PopPUNK compares all possible pairs of genomes by calculating the proportion of shared k-mers of different lengths to determine core and accessory genome distances. It then generates a scatterplot of the two distances to reveal the predicted clustering of isolates^33^.

### In silico molecular typing and detection of AMR genes

ST identification of isolates was confirmed using the program MLST (https://github.com/tseemann/mlst), which extracts the sequences of seven housekeeping genes (*tuf, cpn60*, *pta*, *purA*, *fdh*, *ack*, *sar*^65^) from the contig files and compares them with the *S. pseudintermedius* MLST database (https://pubmlst.org/spseudintermedius/). We screened all of the genomes for known accessory element resistance genes using two programs. For the New England data set, we used a direct read mapping approach implemented in ARIBA^66^ that can identify resistance determinants owing to allelic variants and horizontally acquired AMR genes. For the combined New England and Texas genomes, we used the contig-based search method ABRicate v.0.8.13 (https://github.com/tseemann/abricate). The resistance allele sequences used for comparison were retrieved from the CARD database^67^. A query gene is accepted as a true AMR gene if it reaches a threshold of 95% sequence identity and 95% coverage when compared to the database. We used the default parameters for each program.

### Detection of virulence genes and SCC*mec*

We screened all New England genomes for known virulence genes using ARIBA to query the Virulence Factor Database (VFDB)^68^. Genomes carrying the *mecA*-carrying chromosomal cassette SCC*mec* were identified using SCCmecFinder v.1.2^26^ with minimum thresholds of >60% for sequence coverage and >90% sequence identity. We used the default parameters for each program.

### Estimating recombination rates

We used three approaches to detect recombination in *S. pseudintermedius*. First, we calculated the PHI to determine the statistical likelihood of recombination being present in our data set^36^. If recombination is absent, the genealogical correlation or similarity of neighboring sites remains unchanged to permutations of the sites as all sites have identical evolutionary history^36^. We then visualized potential recombination events using Splitstree v.4.14.6, which shows the presence of conflicting phylogenetic signals for different genes^37^. Second, we ran fastGEAR^38^ with default parameters to detect evidence for recombination in core genes and shared accessory genes. FastGEAR first infers the population structure of individual sequence alignments using a Hidden Markov Model to identify lineages, which are groups of strains that are genetically distinct in at least 50% of the alignment^38^. Within each lineage, recombinations are identified by determining sequence similarity between the target sequence and all other remaining lineages^38^. Third, we used mcorr with default parameters to calculate the correlation profile and different evolutionary parameters using the core gene alignment as input and with 1000 bootstrapped replicates^44^. Mcorr estimates six evolutionary parameters: *θ* – mutational divergence; *ϕ* – recombinational divergence; *c* – recombination coverage or proportion of sites in the genome whose diversity was derived from outside the sample through recombination; *d* – diversity; mean fragment size (*f̅*) of a recombination event; and *θ*/*ϕ* (or *γ*/*μ*) – relative rate of recombination to mutation^44^.

### Statistics and reproducibility

We used Welch's *t* test and *z* score test to compare the different genomic components between the New England and Texas populations. Significance of the inferred PHI was obtained using a permutation test^36^. We used a diversity test implemented in fastGEAR to determine the significance of the inferred recombinations and uncover false positives^38^. We used Welch's *t* test to compare the two *S. pseudintermedius* populations for each of the six parameters calculated by mcorr^44^. A *p* value of <0.05 was considered statistically significant. We used the default parameters for all programs used unless otherwise stated and are then described. Exact sample sizes for each group were described. Source data used to plot Figs. 2d–g, 3a, b, 4b–h are archived in Supplementary Data 9.

### Reporting summary

Further information on research design is available in the Nature Research Reporting Summary linked to this article.

## Supplementary information

Supplementary Information

Description of Additional Supplementary Files

Supplementary Data 1

Supplementary Data 2

Supplementary Data 3

Supplementary Data 4

Supplementary Data 5

Supplementary Data 6

Supplementary Data 7

Supplementary Data 8

Supplementary Data 9

Reporting Summary

## Data Availability

All supporting data are included in this published article and its supplementary material, and are available from the corresponding author upon request. Genome sequence data of the New England samples have been deposited in the NCBI Sequence Read Archive under BioProject accession number PRJNA563147 with BioSample accession numbers listed in Supplementary Data 1. Allelic profiles of the 79 STs previously unidentified to our knowledge from the New England genomes were submitted to the *S. pseudintermedius* database in the MLST website (https://pubmlst.org/spseudintermedius/). Accession numbers of the Texas genomes^32^ are listed in Supplementary Data 1 and have been downloaded from the PATRIC database (https://patricbrc.org). Source data underlying plots shown in figures are provided in Supplementary Data 9.

## Code availability

All tools and R packages used for the analysis are publicly available and fully described in the “Method” sections.
