# Supplementary material for: Population genomics of Staphylococcus pseudintermedius in companion animals in the United States
Source: Commun Biol. 2020 Jun 5;3:282. doi: 10.1038/s42003-020-1009-y (PMC7275049; doi:10.1038/s42003-020-1009-y)
Supplement: Supplementary file 1 — Supplementary Information [file 42003_2020_1009_MOESM1_ESM.pdf]

Population genomics of *Staphylococcus pseudintermedius* in companion animals in the United States

Supplementary figures

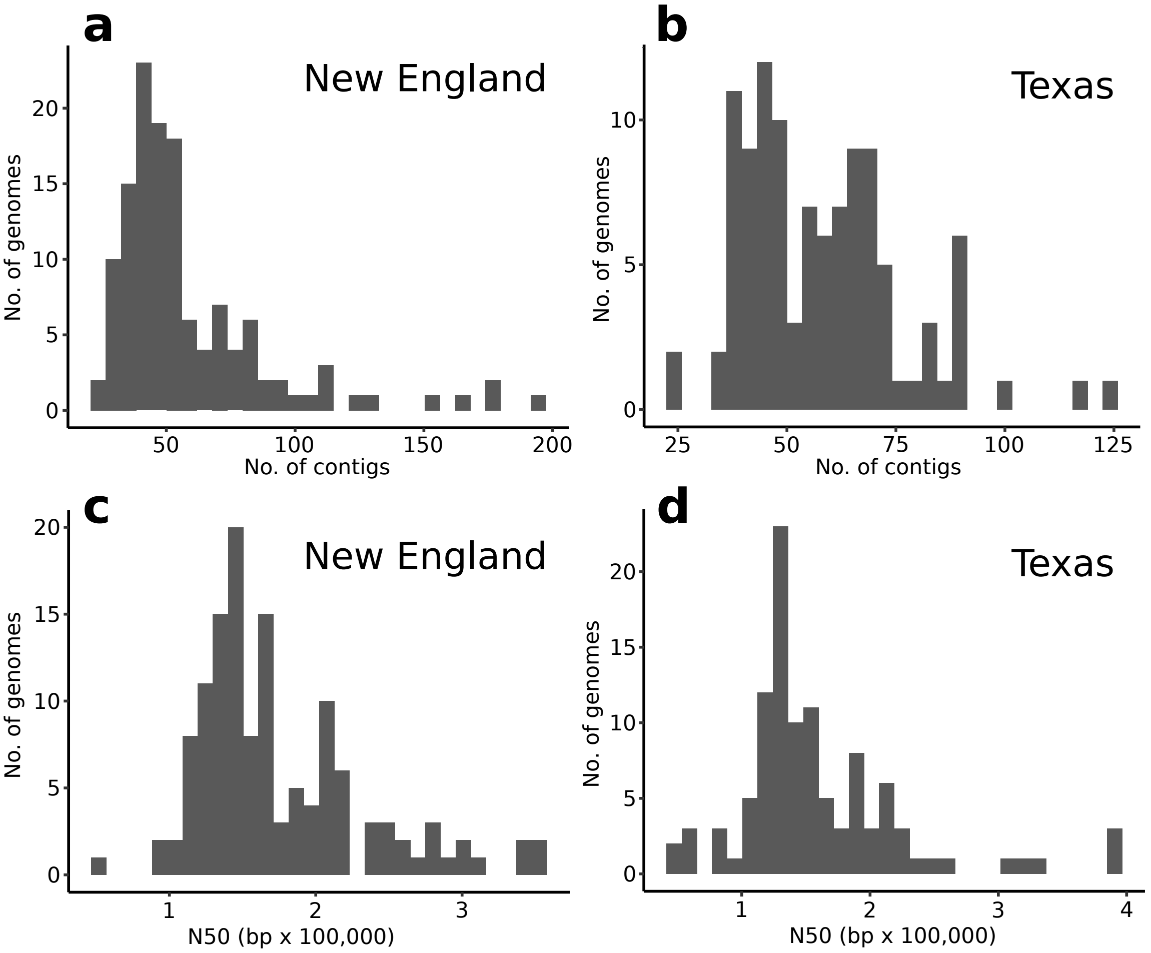

Supplementary Figure 1. Distribution of number of contigs and N50. Histograms indicating contigs per genome for (a) New England and (b) Texas genomes; and N50 values for (c) New England and (d) Texas genomes.

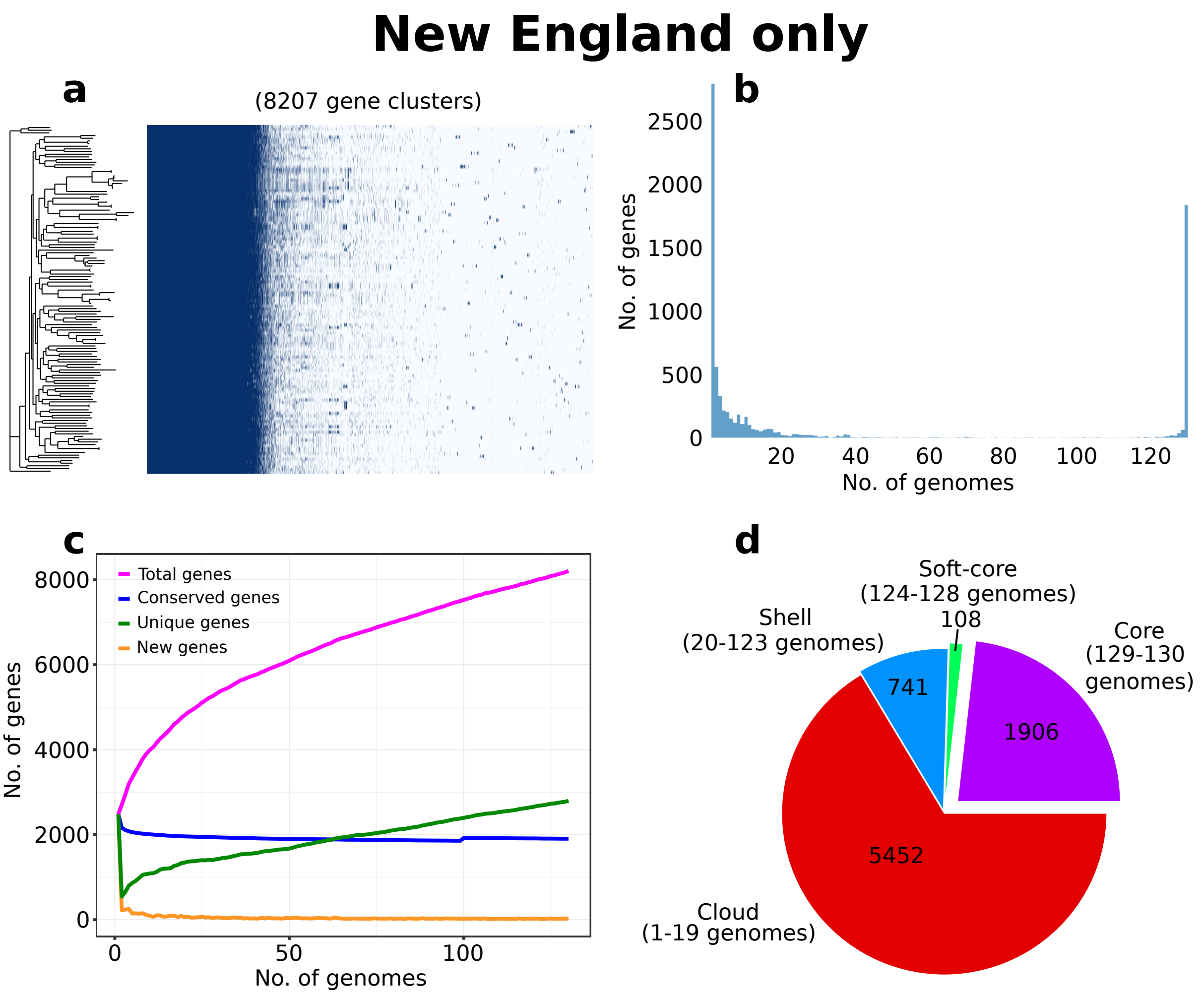

Supplementary Figure 2. Pan-genome analyses of New England *S. pseudintermedius* (n = 130 genomes). (a) Matrix showing gene presence or absence per genome. Each row corresponds to a genome on the phylogeny. Each column represents a unique gene family. (b) The number of unique genes that are shared by any given number of genomes or unique to a single genome. (c) The size of the pan-genome, i.e., the totality of unique genes present in the population (pink line), the size of the core genome, i.e., genes that are present in at least 99% of the strains (blue line), the number of unique genes, i.e., genes unique to an individual strain (green line), and new genes, i.e., genes not found in the previously compared genomes (orange line) in relation to numbers of genomes compared. (d) Pie chart showing the distribution of core, soft core, shell and cloud genes.

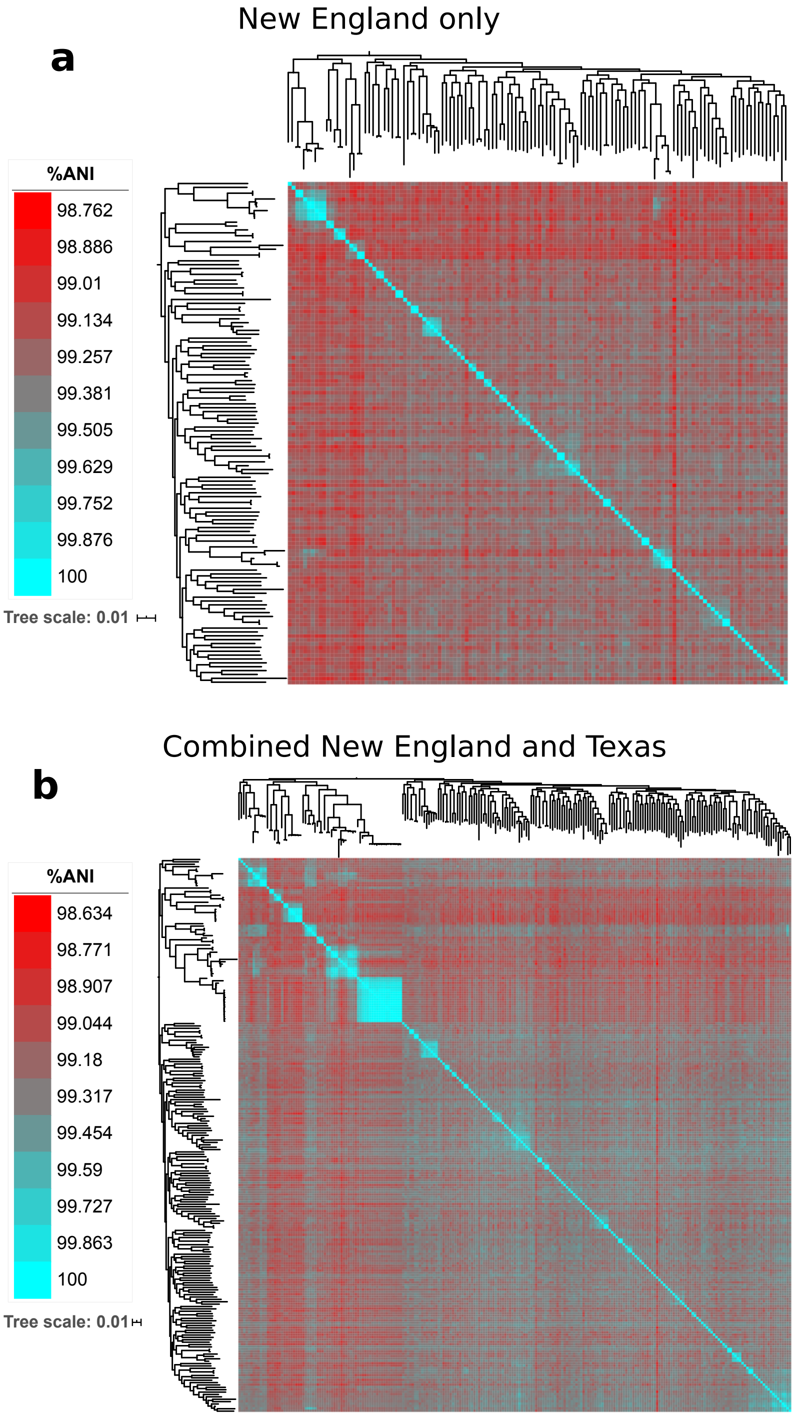

Supplementary Figure 3. Heatmap matrix showing pairwise ANI comparison among all New England genomes (a) and among New England and Texas genomes (b).

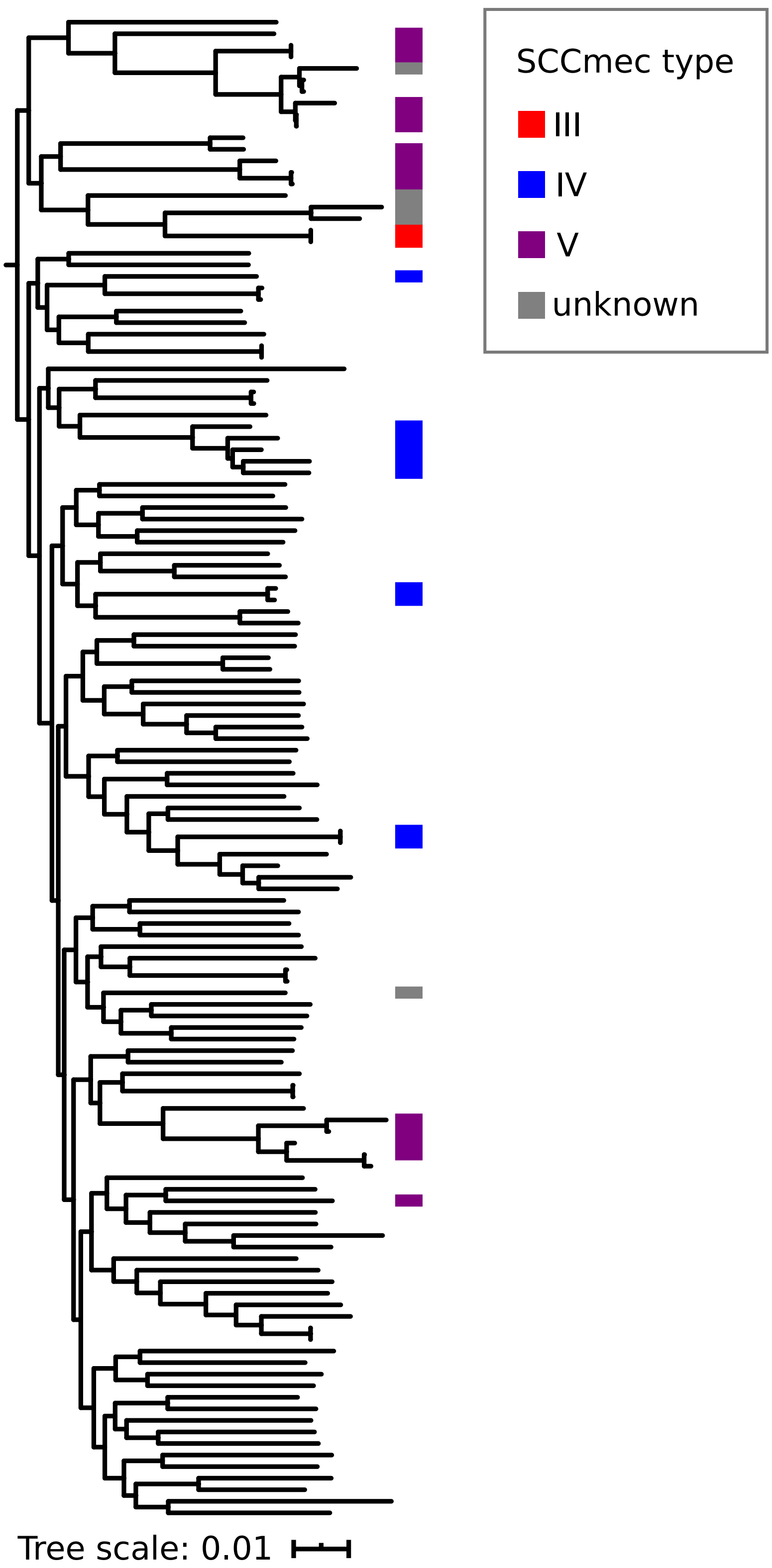

Supplementary Figure 4. Distribution of SCC*mec* types in the New England population.

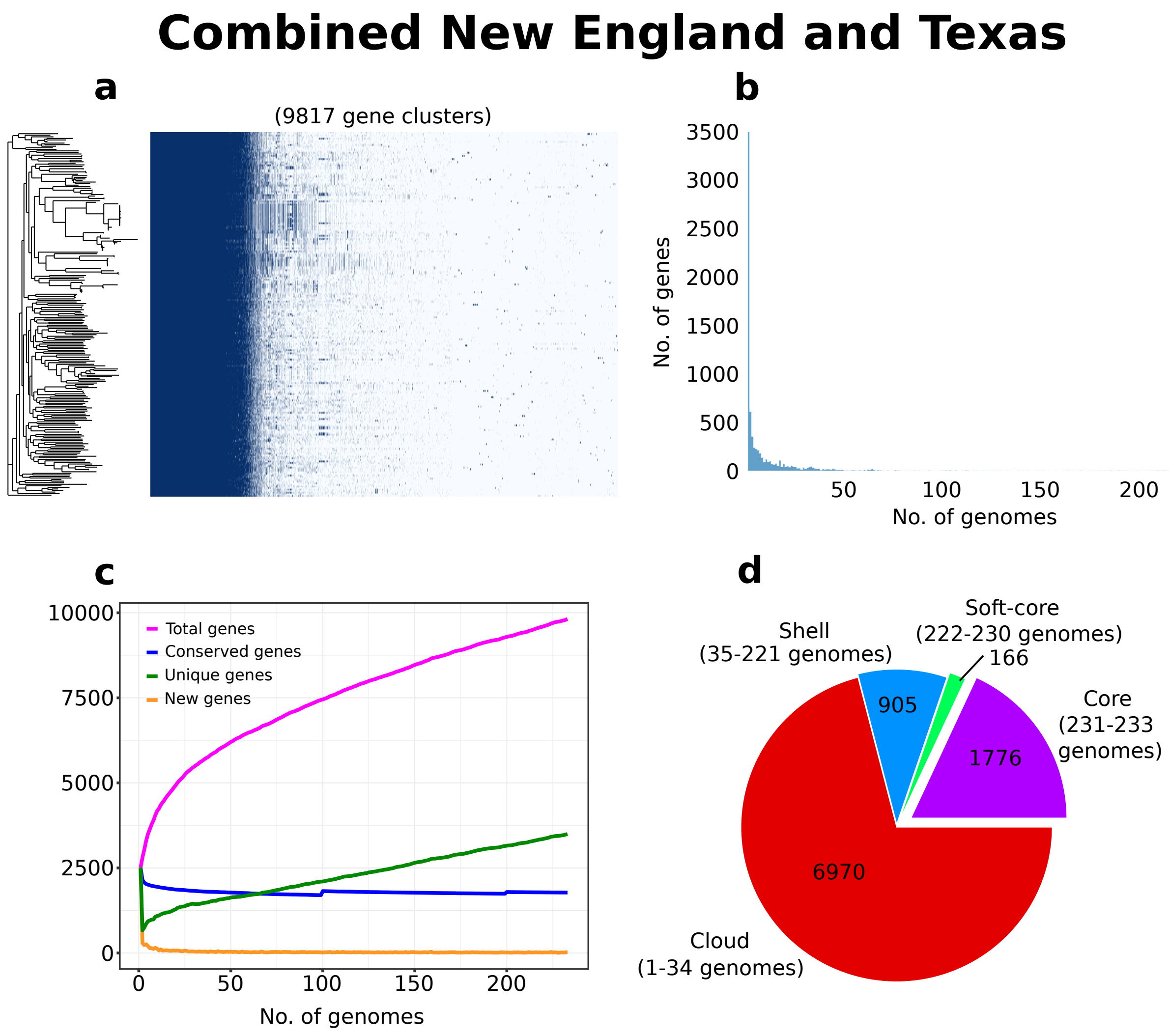

Supplementary Figure 5. Pan-genome analyses of the combined New England and Texas genomes (126 New England and 107 Texas genomes). These are all from dogs. Color legend and definitions are identical to those described in Supplementary Figure 2.
