# Supplementary material for: Population genomics of Staphylococcus pseudintermedius in companion animals in the United States
Source: Commun Biol. 2020 Jun 5;3:282. doi: 10.1038/s42003-020-1009-y (PMC7275049; doi:10.1038/s42003-020-1009-y)
Supplement: Supplementary file 2 — Description of Additional Supplementary Files [file 42003_2020_1009_MOESM2_ESM.pdf]

Supplementary Data

Supplementary Data 1. Accession numbers, metadata and genome characteristics of the 130 New England *S. pseudintermedius* genomes sequenced in this study and the 107 previously published genomes from Texas.

Supplementary Data 2. List of genes (core and accessory genes) identified by Roary in New England genomes (n = 130 genomes), Texas genomes (n = 107 genomes), and in the combined New England and Texas genomes (n = 233 genomes).

Supplementary Data 3. Pairwise ANI values (%) in New England genomes and in the combined New England and Texas genomes. These numbers correspond to Fig. S3.

Supplementary Data 4. Distribution of ABR genes in New England genomes identified by ARIBA and CARD database. This list includes horizontally acquired ABR genes and resistance alleles due to chromosomal mutations.

Supplementary Data 5. Distribution of virulence genes in New England *S. pseudintermedius* genomes identified by ARIBA and VFDB database

Supplementary Data 6. Distribution of ABR genes in Texas genomes identified by ABRicate and CARD database. This list includes only horizontally acquired ABR genes

Supplementary Data 7. List of recombined genes in New England and Texas genomes inferred by fastGEAR

Supplementary Data 8. Evolutionary and recombination parameters that characterize New England and Texas genomes inferred by mcorr.

Supplementary Data 9. Source data for Figures 2d-g, 3a-b, 4b-h
