# Supplementary material for: Population genomics of Staphylococcus pseudintermedius in companion animals in the United States
Source: Commun Biol. 2020 Jun 5;3:282. doi: 10.1038/s42003-020-1009-y (PMC7275049; doi:10.1038/s42003-020-1009-y)
Supplement: Supplementary file 12 — Reporting Summary [file 42003_2020_1009_MOESM12_ESM.pdf]

nature research

Corresponding author(s): Cheryl Andam

Last updated by author(s): Apr 13, 2020

Reporting Summary

Nature Research wishes to improve the reproducibility of the work that we publish. This form provides structure for consistency and transparency in reporting. For further information on Nature Research policies, see Authors & Referees and the Editorial Policy Checklist.

Statistics

For all statistical analyses, confirm that the following items are present in the figure legend, table legend, main text, or Methods section.

n/a	Confirmed	
☐	☒	The exact sample size (*n*) for each experimental group/condition, given as a discrete number and unit of measurement
☐	☒	A statement on whether measurements were taken from distinct samples or whether the same sample was measured repeatedly
☐	☒	The statistical test(s) used AND whether they are one- or two-sided<br>*Only common tests should be described solely by name; describe more complex techniques in the Methods section.*
☒	☐	A description of all covariates tested
☐	☒	A description of any assumptions or corrections, such as tests of normality and adjustment for multiple comparisons
☐	☒	A full description of the statistical parameters including central tendency (e.g. means) or other basic estimates (e.g. regression coefficient) AND variation (e.g. standard deviation) or associated estimates of uncertainty (e.g. confidence intervals)
☐	☒	For null hypothesis testing, the test statistic (e.g. *F*, *t*, *r*) with confidence intervals, effect sizes, degrees of freedom and *P* value noted<br>*Give P values as exact values whenever suitable.*
☒	☐	For Bayesian analysis, information on the choice of priors and Markov chain Monte Carlo settings
☒	☐	For hierarchical and complex designs, identification of the appropriate level for tests and full reporting of outcomes
☒	☐	Estimates of effect sizes (e.g. Cohen's *d*, Pearson's *r*), indicating how they were calculated

Our web collection on statistics for biologists contains articles on many of the points above.

Software and code

Policy information about availability of computer code

Data collection	No software was used for data collection.
Data analysis	Reads were assembled into contigs using the de novo assembler SPAdes v.3.13.1. The resulting contigs were annotated using Prokka. we calculated the genome-wide ANI for all possible pairs of genomes using the program FastANI v.1.0 . We also confirmed the species identity using a BLASTN search of the NCBI non-redundant (nr) database using the annotated 16S rDNA sequences of each genome in our study. Genome assembly quality was assessed using Quast and CheckM. We used the program Roary to characterize the pan-genome. a maximum-likelihood phylogeny was then generated using the program RAxML v.8.2.11. Genetic population structure analysis was performed using R-implemented hierarchical Bayesian analysis of population structure (RhierBAPS). We used PopPUNK (Population Partitioning Using Nucleotide K-mers) to elucidate the elucidate the population genomic structure. ST identification of isolates was confirmed using the program mlst. We screened all of the genomes for known accessory element resistance genes using a direct read mapping approach implemented in ARIBA and the contig-based search method ABRicate v.0.8.13 (https://github.com/tseemann/abricate). Genomes carrying the mecA-carrying chromosomal cassette SCCmec were identified using SCCmecFinder v.1.2. We used PLasmidFinder to identify plasmids in genome sequences. We then visualized potential recombination events using Splitstree v.4.14.6. We ran fastGEAR with default parameters to detect evidence for genome-wide recombination. We used mcorr to calculate the correlation profile and different recombination parameters.

For manuscripts utilizing custom algorithms or software that are central to the research but not yet described in published literature, software must be made available to editors/reviewers. We strongly encourage code deposition in a community repository (e.g. GitHub). See the Nature Research guidelines for submitting code & software for further information.

Data

Policy information about availability of data

All manuscripts must include a data availability statement. This statement should provide the following information, where applicable:

- Accession codes, unique identifiers, or web links for publicly available datasets
- A list of figures that have associated raw data
- A description of any restrictions on data availability

Genome sequence data of the New England samples have been deposited in the NCBI Sequence Read Archive under BioProject accession number PRJNA563147 with BioSample accession numbers listed in Table S1. Allelic profiles of the 79 novel STs from the New England genomes were submitted to the S. pseudintermedius database in the MLST website (https://pubmlst.org/spseudintermedius/). Accession numbers of the Texas genomes 32 are listed in Supplementary Table 1 and have been downloaded from the PATRIC database (https://patricbrc.org).

Field-specific reporting

Please select the one below that is the best fit for your research. If you are not sure, read the appropriate sections before making your selection.

☒ Life sciences ☐ Behavioural & social sciences ☐ Ecological, evolutionary & environmental sciences

For a reference copy of the document with all sections, see nature.com/documents/nr-reporting-summary-flat.pdf

Life sciences study design

All studies must disclose on these points even when the disclosure is negative.

Sample size	Sample size is based on the number of bacterial isolates from clinical animal specimens received by New Hampshire Veterinary Diagnostic Laboratory. The number of genomes analyzed is based on the number of high quality sequences obtained. We also downloaded previously published genome sequences from the PATRIC database and these were included in our analyses.
Data exclusions	No data were excluded from the analyses.
Replication	All genome sequences generated in this study have been deposited in NCBI. Parameters used for each program are described in the Methods. Hence, replication of all analyses and output can be achieved.
Randomization	Randomization is not relevant because all genomes that were sequenced were included in the analyses.
Blinding	Blinding is not relevant because all bacterial isolates and genomes were included in the analyses.

Reporting for specific materials, systems and methods

We require information from authors about some types of materials, experimental systems and methods used in many studies. Here, indicate whether each material, system or method listed is relevant to your study. If you are not sure if a list item applies to your research, read the appropriate section before selecting a response.

Materials & experimental systems

n/a	Involved in the study	
☒	☐	Antibodies
☒	☐	Eukaryotic cell lines
☒	☐	Palaeontology
☐	☒	Animals and other organisms
☒	☐	Human research participants
☒	☐	Clinical data

Methods

n/a	Involved in the study	
☒	☐	ChIP-seq
☒	☐	Flow cytometry
☒	☐	MRI-based neuroimaging

Animals and other organisms

Policy information about studies involving animals; ARRIVE guidelines recommended for reporting animal research

Laboratory animals	The study did not involve laboratory animals.
Wild animals	The study did not involve wild animals.
Field-collected samples	The study did not include animals collected from the field.
Ethics oversight	Although the bacterial isolates came from dogs and cats, no ethical approval was needed because the bacterial isolates were

Ethics oversight	received by New Hampshire Veterinary Diagnostic Laboratory (NHVDL) from routine clinical specimen submissions to NHVDL, which were then forwarded to the laboratory of the senior author.

Note that full information on the approval of the study protocol must also be provided in the manuscript.
